# Complete Genome Sequences of Staphylococcus argenteus Tokyo13064 and Tokyo13069, Isolated from Specimens Obtained during a Food Poisoning Outbreak in Tokyo, Japan

**DOI:** 10.1128/MRA.01447-20

**Published:** 2021-03-11

**Authors:** Yasunori Suzuki, Hiroaki Kubota, Tsutomu Kakuda, Shinji Takai, Kenji Sadamasu

**Affiliations:** a Laboratory of Animal Hygiene, Kitasato University School of Veterinary Medicine, Aomori, Japan; b Department of Microbiology, Tokyo Metropolitan Institute of Public Health, Tokyo, Japan; University of Rochester School of Medicine and Dentistry

## Abstract

The complete genome sequences of two Staphylococcus argenteus strains, Tokyo13064 and Tokyo13069, isolated from human feces and suspected causative foods during a staphylococcal food poisoning outbreak, consist of 2,750,811-bp and 2,751,556-bp circular chromosomes and 2,543 and 2,548 genome annotation-predicted coding DNA sequences, respectively, with 19 rRNAs, 61 tRNAs, and 1 CRISPR each.

## ANNOUNCEMENT

Staphylococcus argenteus is a new species that subdivided from Staphylococcus aureus in 2014 (1). The species epithet of *S. argenteus* reflects the color of its colony (silver) against that of S. aureus (golden) because it is typified by the absence of the carotenoid pigment staphyloxanthin (2, 3). *S*. *argenteus* was previously known as S. aureus clonal complex 75 (CC75) when using multilocus sequence typing (MLST) (4). However, *S*. *argenteus* MLST profiles are not restricted to CC75, and current MLST data subdivide the population structure into at least 5 CCs, including more than 60 sequence types (5).

Recently, *S*. *argenteus* clinical isolates have been reported (6–8), including the first case of food poisoning (an outbreak in Tokyo in 2010) isolates, Tokyo13064 and Tokyo13069, which we reported (9). Tokyo13064 and Tokyo13069 were isolated from feces samples from a patient and suspected causative foods (eggplant, minced meat, and cheese), respectively (9). This report presents a draft genome analysis of Tokyo13064 and Tokyo13069 using MiSeq data and demonstrates the possession or productivity of the causative toxin, namely, enterotoxin. Both *S*. *argenteus* isolates expressed significant levels of staphylococcal enterotoxin B. However, the full-length genome sequences of these strains were not determined. The whole-genome information will expand our understanding of the pathogenicity of this bacterium.

Two *S. argenteus* strains were cultured using brain heart infusion broth overnight at 37°C. Genomic DNA was extracted for sequence analysis using a QIAamp DNA minikit (Qiagen GmbH, Hilden, Germany). The complete genome sequence was obtained by combining sequencing data from both MiSeq (Illumina, San Diego, CA, USA) and MinION (Oxford Nanopore Technologies, Oxford, UK) sequencers. MiSeq data for Tokyo13064 (SRA number DRR083591) and Tokyo13069 (SRA number DRR083592) reported previously (9) are used again in this paper. Illumina sequencing was performed as described in the past (9); specifically, an index-tagged library was prepared using a Nextera XT DNA library preparation kit (Illumina), and 300-bp paired-end reads were sequenced on an Illumina MiSeq instrument. Nanopore sequencing was performed according to the manufacturer’s instructions. Briefly, a DNA library was constructed using a ligation sequencing kit and a native barcoding expansion 1-12 kit (Oxford Nanopore Technologies), and the prepared library was subsequently loaded into a MinION flow cell (R9.4.1; Oxford Nanopore Technologies). The MinION sequencing run was performed for more than 48 h. Base calling and barcoding were performed using Guppy v2.3.7 (Oxford Nanopore Technologies). MiSeq raw reads were trimmed using Trim Galore v0.4.3 (https://www.bioinformatics.babraham.ac.uk/projects/trim_galore/) with default settings, and MinION raw reads were trimmed with NanoFilt v2.6.0 (10) using the following conditions: the reads were discarded if the average quality was less than 10 and the size was less than 500 bp. Hybrid assembly of the MiSeq- and MinION-trimmed reads was performed using Unicycler v0.4.8 with the default parameters (11). Genome error correction, circularization, and rotation were implemented in the Unicycler pipeline. The complete genome sequence was annotated using DFAST v1.2.6 with the default parameters (https://dfast.nig.ac.jp/) (12). Assembly statistics, general genome information, and relevant characteristics are summarized in Table 1.

**TABLE 1:** Assembly statistics, general genome information, and relevant characteristics of Staphylococcus argenteus strains isolated from specimens obtained during the 2010 SFP^*a*^ outbreak in Tokyo, Japan

Strain and genome information	Data for Staphylococcus argenteus strain:	
	Tokyo13064	Tokyo13069
Strain information		
Origin	Feces sample obtained from a patient with SFP during the 2010 outbreak that occurred in Tokyo	Samples of suspected causative foods (eggplant, minced meat, and cheese) of the 2010 SFP outbreak that occurred in Tokyo
Yr of isolation	2010	2010
Sequence type	ST1223	ST1223
Assembly and genome statistics		
MinION		
No. of reads	340,434	364,342
Total no. of bases	2,557,899,489	2,801,243,235
Trimmed with NanoFilt		
No. of reads	273,254	293,628
Read length *N*_50_ (bp)	14,541	14,451
Total no. of bases	2,145,037,520	2,351,940,746
MiSeq		
No. of reads	2,766,350	2,844,530
Total no. of bases	717,210,625	785,402,614
Trimmed with Trim Galore		
No. of reads	2,766,350	2,844,530
Total no. of bases	626,684,460	673,672,330
Coverage (×)	1,007.60	1,099.60
Chromosome description^*b*^		
Genome size (bp)	2,750,811	2,751,556
G+C content (%)	32.4	32.4
No. of CDSs^*c*^	2,543	2,548
Coding ratio (%)	83.5	83.5
No. of rRNAs	19	19
No. of tRNAs	61	61
No. of CRISPRs	1	1

a SFP, staphylococcal food poisoning.

b All genomic statistics are output from the DFAST pipeline.

c CDSs, coding sequences.

### Data availability.

The complete genome sequences of these two *S*. *argenteus* strains, Tokyo13064 and Tokyo13069, have been deposited in the DNA Data Bank of Japan (DDBJ)/GenBank under accession numbers AP024200 (Tokyo13064) and AP024201 (Tokyo13069). The raw MinION read data for each can be found in the DDBJ/NCBI Sequence Read Archive under accession numbers DRR257742 (Tokyo13064) and DRR257743 (Tokyo13069).
